# The role of PSMC4 in non-small cell lung cancer: implications for prognosis, diagnosis, and immune microenvironment modulation

**DOI:** 10.3389/fonc.2025.1503466

**Published:** 2025-05-02

**Authors:** Lili Zhu, Yuanjun Li, Yunfei Xu, Jian Lei

**Affiliations:** ^1^ Department of Pathology, Hunan Cancer Hospital, The Affiliated Cancer Hospital of Xiangya School of Medicine, Central South University, Changsha, Hunan, China; ^2^ Department of Physiology, School of Basic Medical Science, Central South University, Changsha, Hunan, China; ^3^ Postdoctoral Research Station of Biology, School of Basic Medical Science, Central South University, Changsha, Hunan, China

**Keywords:** PSMC4, non-small cell lung cancer, prognosis, immune infiltration, cell proliferation

## Abstract

**Introduction:**

Non-small cell lung cancer (NSCLC) remains a principal cause of cancer-related mortality. The discovery of novel biomarkers is pivotal for enhancing early diagnosis, refining prognostic evaluations, and optimizing targeted therapeutic strategies. Proteasome 26S subunit ATPase 4 (PSMC4), a proteasome subunit essential for protein degradation, influences tumor progression regulatory mechanisms. Despite its recognized importance, the specific contributions of PSMC4 to NSCLC progression are not well defined.

**Methods:**

This investigation employs a combination of bioinformatics and histological methods to delineate the expression profile of PSMC4 in NSCLC and its correlations with clinicopathological characteristics, diagnostic efficacy, prognostic value, and tumor microenvironment.

**Results:**

We reveal an elevated level of PSMC4 in various malignancies, notably lung adenocarcinoma. Elevated levels of PSMC4 are strongly associated with higher pathological T stages, N stages, and pathological stages. Analysis using receiver operating characteristic curves demonstrates the high diagnostic sensitivity and specificity of PSMC4. Furthermore, patients with elevated PSMC4 levels experience markedly reduced overall survival, disease-specific survival, and progression-free intervals. Both univariate and multivariate Cox regression analyses substantiate that PSMC4 serves as an independent prognostic marker. Analysis of differentially expressed genes and functional annotation demonstrate that genes related to PSMC4 are crucial across a spectrum of biological processes, including DNA replication, chromatin assembly, and mitotic prophase. Gene set enrichment analysis reveals significant correlations between PSMC4 and essential signaling pathways such as the G2/M DNA damage checkpoint, WNT signaling pathway, and cellular senescence. Moreover, immunohistochemical evaluations confirm the increased expression of PSMC4 in NSCLC tissues. Functional assays reveal that PSMC4 accelerates the proliferation of lung cancer cells and tumor growth in xenograft models.

**Discussion:**

These results highlight the potential of PSMC4 as a diagnostic and prognostic marker in NSCLC and elucidate its integral role within the tumor immune microenvironment. Consequently, targeting PSMC4 emerges as a viable therapeutic approach for NSCLC.

## 1 Introduction

Lung adenocarcinoma (LUAD) is the most common histological subtype of non-small cell lung cancer (NSCLC), which accounts for approximately 85% of all lung cancer cases (1, 2). Lung cancer remains the leading cause of cancer-related mortality worldwide, with an estimated 1.8 million deaths annually (3). Despite advances in diagnostic and therapeutic strategies, the five-year survival rate for lung adenocarcinoma patients remains dismally low, primarily due to late-stage diagnosis and the development of resistance to conventional therapies (4–6). Current treatment modalities, including surgery, chemotherapy, radiotherapy, and targeted therapy, have shown limited efficacy in improving long-term survival, underscoring the urgent need for novel biomarkers and therapeutic targets to enhance early diagnosis and treatment outcomes (7, 8).

The proteasome 26S subunit, ATPase 4 (PSMC4), is a crucial component of the 26S proteasome complex involved in the degradation of ubiquitinated proteins, thereby regulating various cellular processes such as cell cycle progression, apoptosis, and DNA repair (9, 10). Recent studies have implicated PSMC4 in various cancers, including breast cancer (10), endometrial cancer (11, 12), prostate carcinoma (13, 14), and oral squamous cell carcinoma (15), where its dysregulation contributes to tumorigenesis and cancer progression. Research has revealed that elevated expression of PSMC4 correlates positively with reduced survival rates in breast cancer patients (10). Additionally, evidence suggests that PSMC4 may influence the progression of prostate cancer by modulating the CBX3-EGFR-PI3K-AKT-mTOR signaling pathway (14). These findings suggest that PSMC4 may serve as a potential biomarker and therapeutic target in cancer. However, the role of PSMC4 in lung cancer remains largely unexplored.

In this study, we investigated the expression levels of PSMC4 in lung adenocarcinoma and assessed its associations with clinicopathological features, immune cell infiltration, and functional enrichment. Utilizing data from public repositories, including The Cancer Genome Atlas (TCGA), the Genotype-Tissue Expression project (GTEx), and the Gene Expression Omnibus (GEO), we analyzed the expression of PSMC4 and its correlation with clinical outcomes. Considering the pivotal role of immune status in the survival of lung cancer patients (16, 17), we also explored the impact of PSMC4 expression on immune cell infiltration. Furthermore, we conducted comprehensive *in vitro* and *in vivo* experiments to delineate the functional role of PSMC4 in the progression of lung cancer.

## 2 Materials and methods

### 2.1 Data processing and ethical statement

For this investigation, comprehensive gene expression data were sourced from prominent public databases, including The Cancer Genome Atlas (TCGA) from the National Cancer Institute, the Genotype-Tissue Expression (GTEx) project, and the Gene Expression Omnibus (GEO). These repositories provided a robust foundation for analyzing the expression patterns of the PSMC4 gene in lung adenocarcinoma as well as a spectrum of other cancers. We extracted mRNA expression profiles for 33 cancer types and their corresponding normal tissues, primarily from the TCGA database. Additionally, the GEO dataset GSE19804, which comprises expression data from 60 lung cancer specimens alongside 60 control samples, was utilized for comparative analyses.

Data from TCGA were retrieved directly from its official portal (https://portal.gdc.cancer.gov/), encompassing extensive gene expression data and relevant clinical information across 33 cancer types. GTEx data were accessed via its dedicated portal (https://gtexportal.org/), providing a comprehensive reference for normal tissue gene expression. The dataset GSE19804 from GEO was downloaded from its platform (https://www.ncbi.nlm.nih.gov/geo/), offering balanced gene expression data from both lung cancer and normal samples.

Following data acquisition, rigorous procedures—including quality control, normalization, and statistical analyses—were implemented to ensure the integrity and consistency of the data. Given that all samples were derived from publicly accessible databases with the necessary ethical approvals, this study was exempt from further ethical review. Throughout the research, stringent measures were taken to protect participant privacy and rights.

### 2.2 Correlation analysis between PSMC4 gene expression and clinicopathological features in lung adenocarcinoma

To elucidate the association between PSMC4 gene expression and the clinicopathological characteristics of lung adenocarcinoma, we conducted a series of statistical analyses. These included chi-square tests for categorical variables, logistic regression for binary outcomes, and Wilcoxon rank-sum tests for continuous variables. The diagnostic potential of the PSMC4 gene was assessed through Receiver Operating Characteristic (ROC) analysis. Survival probabilities were estimated using Kaplan-Meier curve analysis, while the impact of variables on survival was quantified using both univariate and multivariate Cox proportional hazards regression models. Visualization of prognostic indicators was achieved through the construction of nomograms, utilizing the “rms” package in R for both nomogram creation and calibration plot generation.

### 2.3 Differential gene expression and functional enrichment analysis related to PSMC4 expression levels in lung adenocarcinoma patients

Patients in the TCGA lung adenocarcinoma cohort were stratified into low and high PSMC4 expression groups based on the median expression level of PSMC4 mRNA. Differential gene expression between these groups was analyzed using the “limma” package in R, with results visually represented in volcano plots and ranked difference plots to highlight the significant contrasts. For further insight into the biological implications of these expression differences, functional enrichment analyses were performed. This included assessing Gene Ontology (GO) categories, Kyoto Encyclopedia of Genes and Genomes (KEGG) pathways, and Gene Set Enrichment Analysis (GSEA), using the “clusterProfiler” (18) package in R to identify enriched pathways and biological processes associated with the dichotomized expression of PSMC4.

### 2.4 Exploration of the association between PSMC4 and immune cell infiltration in lung adenocarcinoma

To elucidate the link between PSMC4 gene expression and immune cell infiltration in lung adenocarcinoma, we utilized the single-sample Gene Set Enrichment Analysis (ssGSEA) algorithm, as implemented in the “GSVA” (19) package in R. This approach allowed for the quantification of enrichment across 24 prevalent immune cell types. The relationship between PSMC4 expression levels and the respective infiltration scores of these immune cell types was assessed using Spearman correlation analysis.

### 2.5 Patient samples and tissue collection

Patient samples and tissues for this study were obtained from individuals diagnosed with lung cancer at the Hunan Cancer Hospital over the period from 2021 to 2023. All procedural aspects of sample collection were conducted in compliance with ethical standards set forth by the Ethics Committee of Hunan Cancer Hospital (2024101). Informed consent was secured from each participant prior to sample collection.

### 2.6 Immunohistochemical staining

Immunohistochemical analysis was performed in accordance with established protocols and evaluation criteria (20). For the detection of PSMC4, tissues were stained using a rabbit anti-PSMC4 antibody (1:100; Proteintech, Wuhan, China; 11389-1-AP).

### 2.7 Cell culture and gene transfection

Human lung adenocarcinoma cell lines, A549 and H1299, were cultured in RPMI-1640 medium enriched with 10% fetal bovine serum. These cells were maintained at 37°C in a 5% CO_2_ humidified incubator. For studies on gene function, cells underwent transfection with plasmids containing either PSMC4 short hairpin RNA or PSMC4 complementary DNA using Lipofectamine 3000 (Invitrogen), adhering strictly to the manufacturer’s guidelines. Cells were collected 48 hours after transfection for further analysis.

### 2.8 Western blot

Proteins were harvested using RIPA buffer from the cells and quantified with a BCA Protein Assay Kit. Following separation by SDS-PAGE, proteins were transferred to PVDF membranes. These membranes were then probed with a rabbit anti-PSMC4 antibody (1:1000; Proteintech, Wuhan, China; 11389-1-AP) and a mouse anti-GAPDH antibody (1:7000; Proteintech, Wuhan, China; 60004-1-Ig). Detection of protein bands was facilitated by enhanced chemiluminescence detection.

### 2.9 Cell proliferation and colony formation assays

Cell proliferation was evaluated using the CCK-8 assay. After transfection, cells were plated at a density of 5,000 cells per well in 96-well plates. CCK-8 solution (Yeasen Biotechnology, Shanghai, China) was administered every 24 hours, and absorbance was measured with a microplate reader to monitor cell growth. For colony formation assays, cells were seeded in 6-well plates and cultured for two weeks, followed by fixation with methanol and staining with crystal violet to facilitate colony enumeration.

### 2.10 Establishment of mouse tumor models

For the establishment of mouse tumor models, six-week-old nude mice were randomly assigned into groups of two. Human lung adenocarcinoma H1299 cells, transfected with either PSMC4 overexpression plasmids or control plasmids (1×10^6 cells per mouse), were subcutaneously injected into the right flank of each mouse. Tumor growth was monitored every week, with tumor volume calculated using the formula (V = 0.5 × length × width²). After the experiment, mice were euthanized, and tumors were excised, weighed, and subjected to further analysis. All experimental procedures were conducted in accordance with ethical guidelines and approved by the Ethics Committee of Hunan Cancer Hospital (D2021-142).

### 2.11 Statistical analysis

Statistical analyses were performed using SPSS version 20.0 (IBM, Armonk, NY, USA) and GraphPad Prism version 8.0.2 (San Diego, California, USA). We designated a p-value of less than 0.05 as statistically significant. To assess differences in quantitative and categorical data between groups, we applied one-way analysis of variance (ANOVA) and Student’s t-tests for continuous variables, and chi-square tests or Fisher’s exact tests for categorical variables, as appropriate.

## 3 Results

### 3.1 Elevated expression of PSMC4 across diverse cancer types, including lung cancer

**Figure 1:**
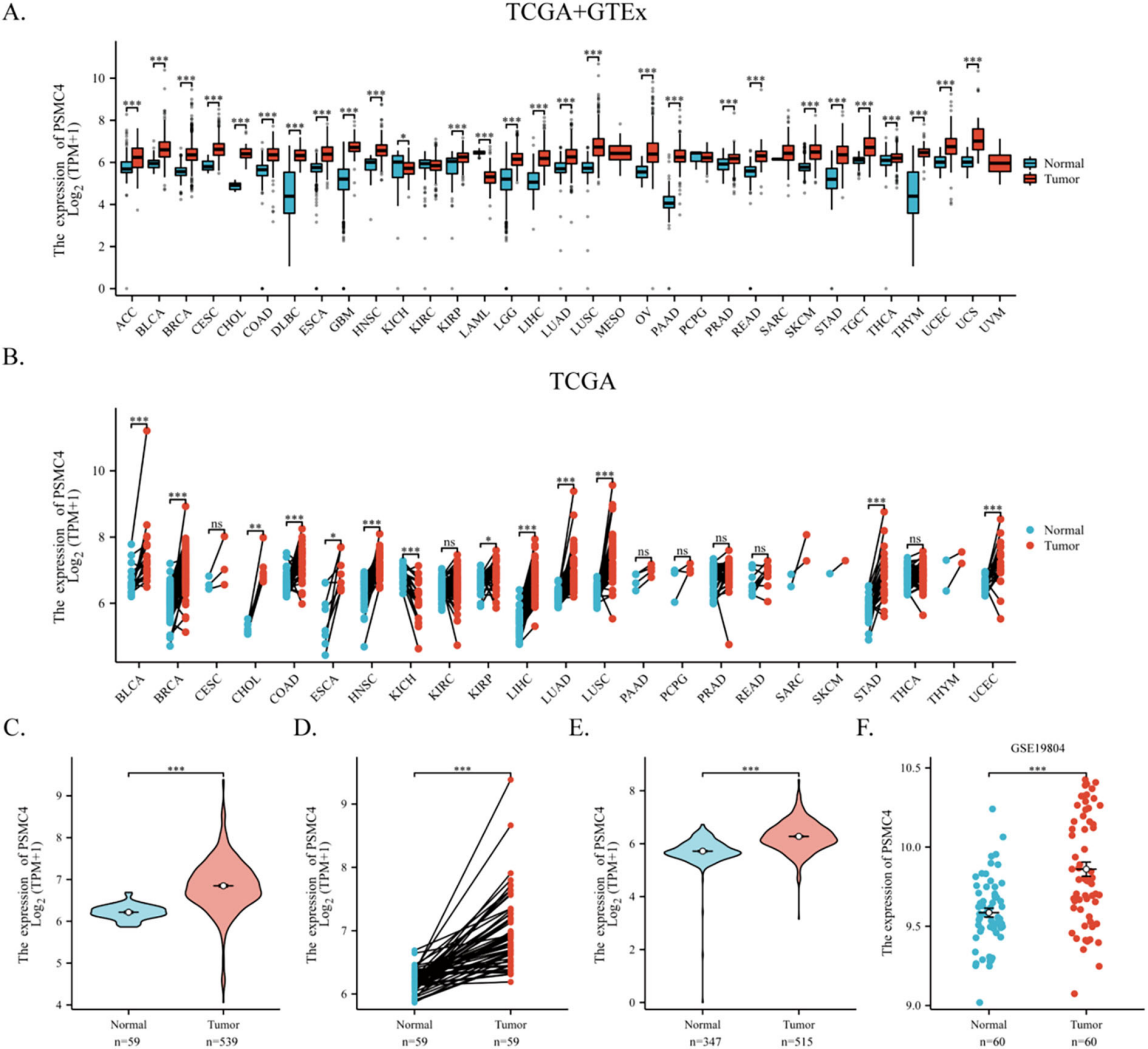
Upregulation of PSMC4 expression in lung cancer. **(A, B)** Expression levels of PSMC4 in various cancer tissues and their adjacent normal tissues from TCGA and GTEx databases. **(C, D)** Comparison of PSMC4 expression levels between normal tissues and lung adenocarcinoma tissues from the TCGA database. **(E)** Comparison of PSMC4 expression levels between lung adenocarcinoma and adjacent normal tissues from TCGA and GTEx databases. **(F)** Analysis of PSMC4 expression levels in the GEO dataset GSE19804 shows significant differences (ns: p>0.05, *p<0.05, **p<0.01, ***p<0.001) (ACC, Adrenocortical carcinoma; BLCA, Bladder urothelial carcinoma; BRCA, Breast invasive carcinoma; CESC, Cervical squamous cell carcinoma and endocervical adenocarcinoma; CHOL, Cholangiocarcinoma; COAD, Colon adenocarcinoma; DLBC, Diffuse large B-cell lymphoma; ESCA, Esophageal carcinoma; GBM, Glioblastoma multiforme; HNSC, Head and neck squamous cell carcinoma; KICH, Kidney chromophobe; KIRC, Kidney renal clear cell carcinoma; KIRP, Kidney renal papillary cell carcinoma; LAML, Acute myeloid leukemia; LGG, Low-grade glioma; LIHC, Liver hepatocellular carcinoma; LUAD, Lung adenocarcinoma; LUSC, Lung squamous cell carcinoma; MESO, Mesothelioma; OV, Ovarian serous cystadenocarcinoma; PAAD, Pancreatic adenocarcinoma; PCPG, Pheochromocytoma and paraganglioma; PRAD, Prostate adenocarcinoma; READ, Rectum adenocarcinoma; SARC, Sarcoma; SKCM, Skin cutaneous melanoma; STAD, Stomach adenocarcinoma; TGCT, Testicular germ cell tumors; THCA, Thyroid carcinoma; THYM, Thymoma; UCEC, Uterine corpus endometrial carcinoma; UCS, Uterine carcinosarcoma; UVM, Uveal melanoma).

Utilizing TCGA dataset and the GTEx database, our comprehensive analysis of PSMC4 mRNA expression across 33 cancer types and their corresponding normal tissues revealed significant upregulation of PSMC4 in 26 out of these 33 types, most notably in hepatocellular carcinoma, lung adenocarcinoma, squamous cell carcinoma of the lung, and gastric cancer (**Figure 1A**). Paired sample analysis robustly confirmed PSMC4’s elevated expression in these malignancies (**Figure 1B**). A detailed examination of 539 lung adenocarcinoma samples and 59 normal lung samples from TCGA highlighted a pronounced increase in PSMC4 expression in lung adenocarcinoma (**Figure 1C**). This elevation was corroborated through differential analysis of paired samples, where lung adenocarcinoma exhibited significantly higher levels of PSMC4 compared to the normal lung tissues (**Figure 1D**). Further integration of data from TCGA and GTEx, encompassing an expanded cohort of 347 normal tissues, also demonstrated a marked upregulation of PSMC4 in LUAD (**Figure 1E**). Moreover, analysis of the GEO dataset GSE19804, which includes 60 lung cancer samples and 60 matched normal samples, consistently supported the upregulation of PSMC4 in lung cancer (**Figure 1F**). These observations collectively underscore the widespread upregulation of PSMC4 across multiple cancer types, emphasizing its potential role in tumorigenesis.

### 3.2 Correlation between PSMC4 expression and clinical pathologic features in lung adenocarcinoma

**Figure 2:**
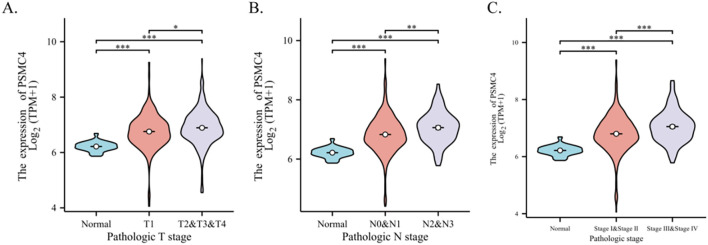
Correlation between PSMC4 expression and clinical pathological features in lung adenocarcinoma patients. The association of PSMC4 expression with T stage **(A)**, N stage **(B)**, and pathologic stage **(C)** in lung adenocarcinoma patients were analyzed using the Wilcoxon rank-sum test (*p<0.05, **p<0.01, ***p<0.001).

Our investigation elucidates the relationship between the expression of PSMC4 and various clinical-pathological characteristics in lung adenocarcinoma. **Table 1** illustrates that patients with elevated PSMC4 expression tend to present with more advanced pathological T stages, N stages, and overall pathologic stages compared to those exhibiting lower expression levels. However, no notable differences were observed in other clinical-pathological parameters. Further stratification of PSMC4 expression against different pathological indices demonstrated a significant positive correlation with the severity of T stage, N stage, and overall pathologic stage (**Figures 2A–C**). Specifically, increased PSMC4 levels were significantly associated with advanced stages in these categories. Logistic regression analysis substantiated this positive association, affirming that higher PSMC4 expression correlates with more advanced T stage, N stage, and pathologic stage in patients with lung adenocarcinoma (**Table 2**). This correlation underscores the potential of PSMC4 as a prognostic marker for tumor progression in lung adenocarcinoma.

**Table 1:** Correlation analysis of PSMC4 expression with clinicopathological features in lung adenocarcinoma patients.

Characteristics	Low expression of PSMC4	High expression of PSMC4	*p-*value
n	269	270	
Gender, n (%)			0.130
Female	153 (56.9%)	136 (50.4%)	
Male	116 (43.1%)	134 (49.6%)	
Age, n (%)			0.660
<= 65	132 (50.4%)	125 (48.4%)	
> 65	130 (49.6%)	133 (51.6%)	
Pathologic T stage, n (%)			0.027
T1	100 (37.3%)	76 (28.4%)	
T2&T3&T4	168 (62.7%)	192 (71.6%)	
Pathologic N stage, n (%)			0.009
N0&N1	231 (89.5%)	216 (81.5%)	
N2&N3	27 (10.5%)	49 (18.5%)	
Pathologic M stage, n (%)			0.247
M0	175 (95.1%)	190 (92.2%)	
M1	9 (4.9%)	16 (7.8%)	
Pathologic stage, n (%)			0.003
Stage I&Stage II	223 (84.5%)	198 (74.2%)	
Stage III&Stage IV	41 (15.5%)	69 (25.8%)	
Smoker, n (%)			0.398
No	42 (16%)	35 (13.4%)	
Yes	221 (84%)	227 (86.6%)	

**Table 2:** Logistic regression model assessing the association between PSMC4 expression and clinicopathological characteristics in lung adenocarcinoma patients.

Characteristics	Total (N)	OR (95% CI)	*p-*value
Gender (Male vs. Female)	539	1.300 (0.926 – 1.825)	0.130
Age (> 65 vs. <= 65)	520	1.080 (0.766 – 1.524)	0.660
Pathologic T stage (T2&T3&T4 vs. T1)	536	1.504 (1.046 – 2.162)	**0.028**
Pathologic N stage (N2&N3 vs. N0&N1)	523	1.941 (1.171 – 3.216)	**0.010**
Pathologic M stage (M1 vs. M0)	390	1.637 (0.705 – 3.801)	0.251
Pathologic stage (Stage III&Stage IV vs. Stage I&Stage II)	531	1.895 (1.231 – 2.917)	**0.004**
Smoker (Yes vs. No)	525	1.233 (0.759 – 2.003)	0.398

Bold values indicate P<0.05.

### 3.3 PSMC4 as a potential diagnostic and prognostic biomarker for lung adenocarcinoma

**Figure 3:**
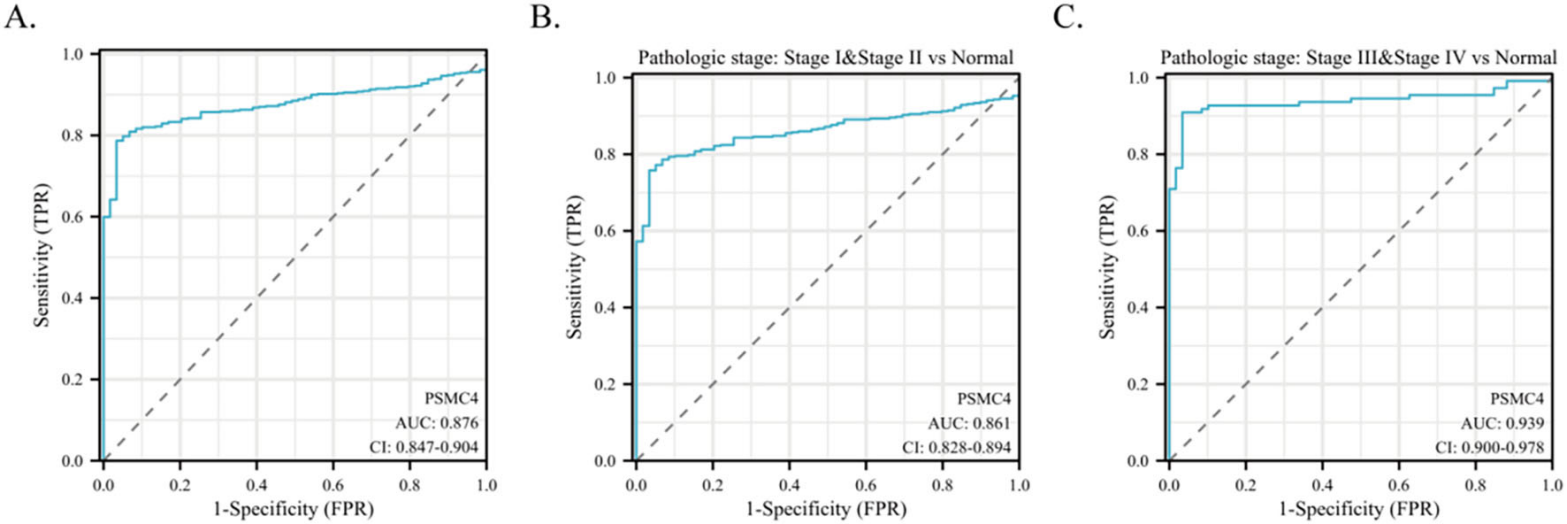
Predictive potential of PSMC4 in lung adenocarcinoma diagnosis. **(A)** ROC curve evaluation of the diagnostic value of PSMC4 in lung adenocarcinoma. **(B, C)** Subgroup analysis based on pathological stages I-II and III-IV.

**Figure 4:**
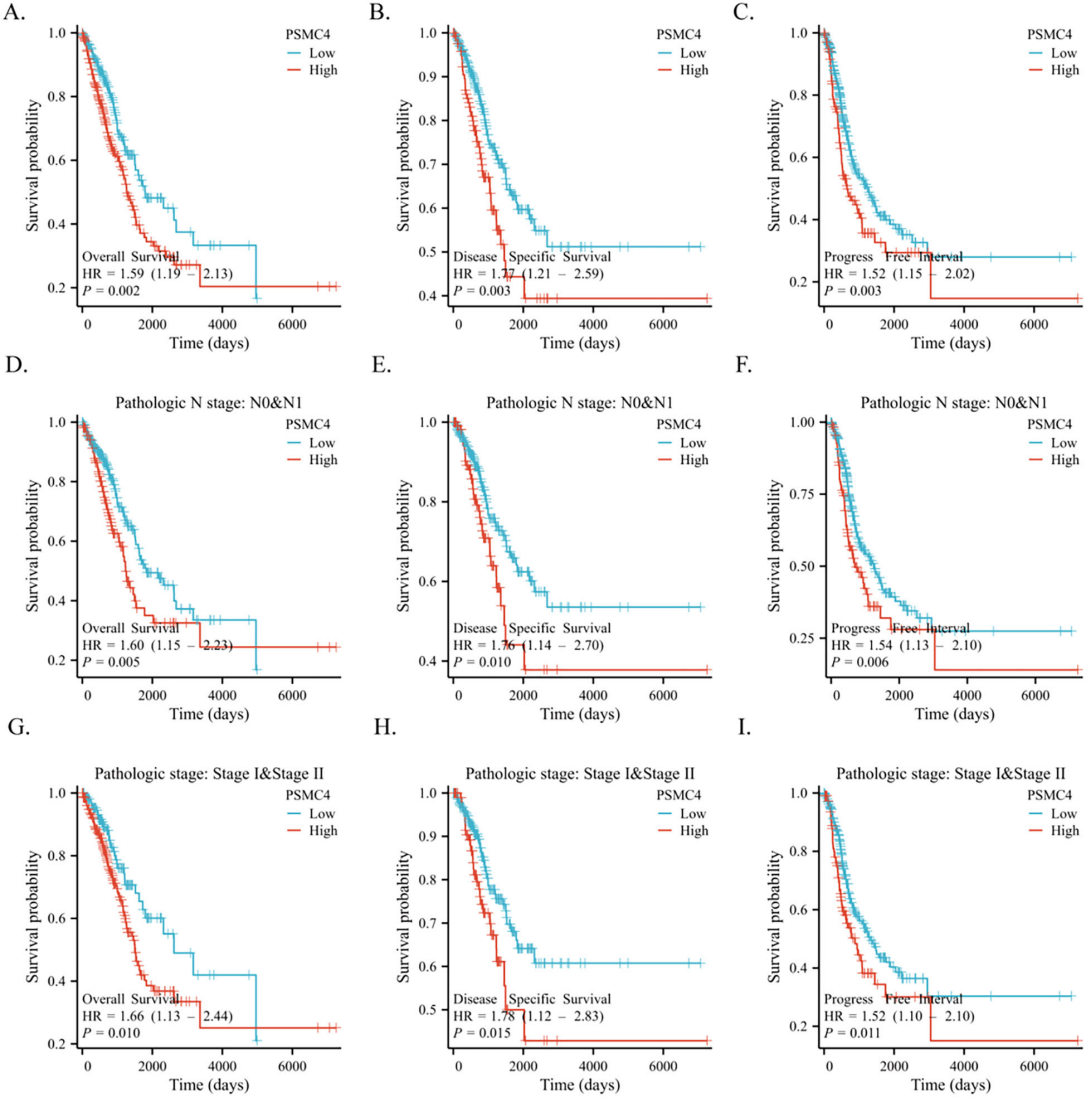
Application of PSMC4 in the clinical prognosis of lung adenocarcinoma. Kaplan-Meier survival analysis comparing high and low PSMC4 expression levels in lung adenocarcinoma patients in terms of overall survival **(A)**, disease-specific survival **(B)**, and progression-free interval **(C)**. Additionally, the same analysis was performed for N0/N1 stage **(D-F)** and pathological stages I-II **(G-I)** lung adenocarcinoma patients.

The diagnostic capabilities of PSMC4 in lung adenocarcinoma were determined through ROC curve analysis, revealing an area under the curve (AUC) of 0.876, which reflects high diagnostic sensitivity and specificity (**Figure 3A**). The diagnostic efficacy of PSMC4 varied with pathological stages, manifesting AUC values of 0.861 for stages I-II and 0.939 for stages III-IV, underscoring its stage-dependent utility (**Figures 3B,C**). Kaplan-Meier survival analysis was employed to ascertain the prognostic significance of PSMC4 expression in clinical outcomes. Notably, elevated PSMC4 expression was associated with significantly reduced overall survival (OS), disease-specific survival (DSS), and progression-free interval (PFI), highlighting its adverse impact on survival metrics (p<0.01) (**Figures 4A–C**). In subgroup analyses, high PSMC4 expression particularly compromised prognoses in patients with early-stage lung adenocarcinoma (N0/N1 and pathological stages I-II), indicating its potential as an early adverse prognostic marker (**Figures 4D–I**).

**Figure 5:**
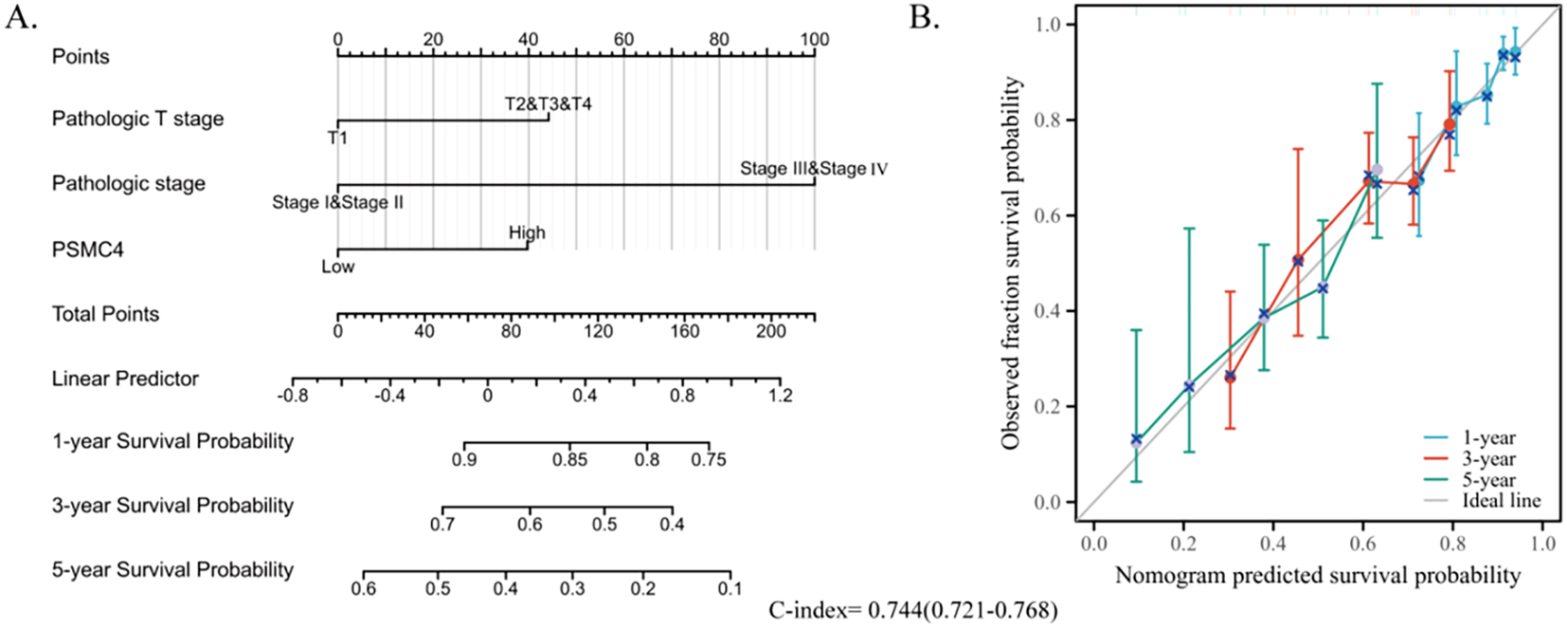
Development of a prognostic nomogram based on PSMC4 expression. **(A)** Nomogram depicting the 1-year, 3-year, and 5-year overall survival risks based on PSMC4 expression. **(B)** Calibration plot validating the accuracy of the nomogram in predicting overall survival.

To further delineate risk factors influencing overall survival, both univariate and multivariate Cox regression analyses were executed. Univariate analysis underscored the association of T stage, N stage, pathologic stage, and PSMC4 expression with poor prognosis (**Table 3**, p<0.05). The multivariate analysis, adjusting for confounders, identified the T stage, pathologic stage, and PSMC4 expression as independent prognostic indicators (p<0.05). Additionally, the prognostic model we developed, which integrates these significant independent factors, demonstrated robust predictive accuracy with a concordance index (c-index) of 0.744, affirming its utility in clinical prognostication (**Figures 5A, B**).

**Table 3:** Cox regression analysis of overall survival in lung adenocarcinoma patients based on clinical outcomes.

Characteristics	Total(N)	Univariate analysis		Multivariate analysis	
		Hazard ratio (95% CI)	*p-*value	Hazard ratio (95% CI)	*p-*value
Gender	530				
Female	283	Reference			
Male	247	1.087 (0.816 - 1.448)	0.569		
Age	520				
<= 65	257	Reference			
> 65	263	1.216 (0.910 - 1.625)	0.186		
Pathologic T stage	527				
T1	176	Reference		Reference	
T2&T3&T4	351	1.717 (1.221 - 2.415)	**0.002**	1.421 (1.004 - 2.011)	**0.048**
Pathologic N stage	514				
N0&N1	441	Reference		Reference	
N2&N3	73	2.360 (1.659 - 3.358)	**< 0.001**	0.913 (0.524 - 1.592)	0.749
Pathologic stage	522				
Stage I&Stage II	415	Reference		Reference	
Stage III&Stage IV	107	2.710 (1.994 - 3.685)	**< 0.001**	2.538 (1.554 - 4.143)	**< 0.001**
Smoker	516				
No	74	Reference			
Yes	442	0.942 (0.625 - 1.420)	0.775		
PSMC4	530				
Low	266	Reference		Reference	
High	264	1.589 (1.187 - 2.127)	**0.002**	1.412 (1.043 - 1.911)	**0.026**

Bold values indicate P<0.05.

### 3.4 Comparative analysis of differentially expressed genes in lung adenocarcinoma patients with varying PSMC4 expression levels

**Figure 6:**
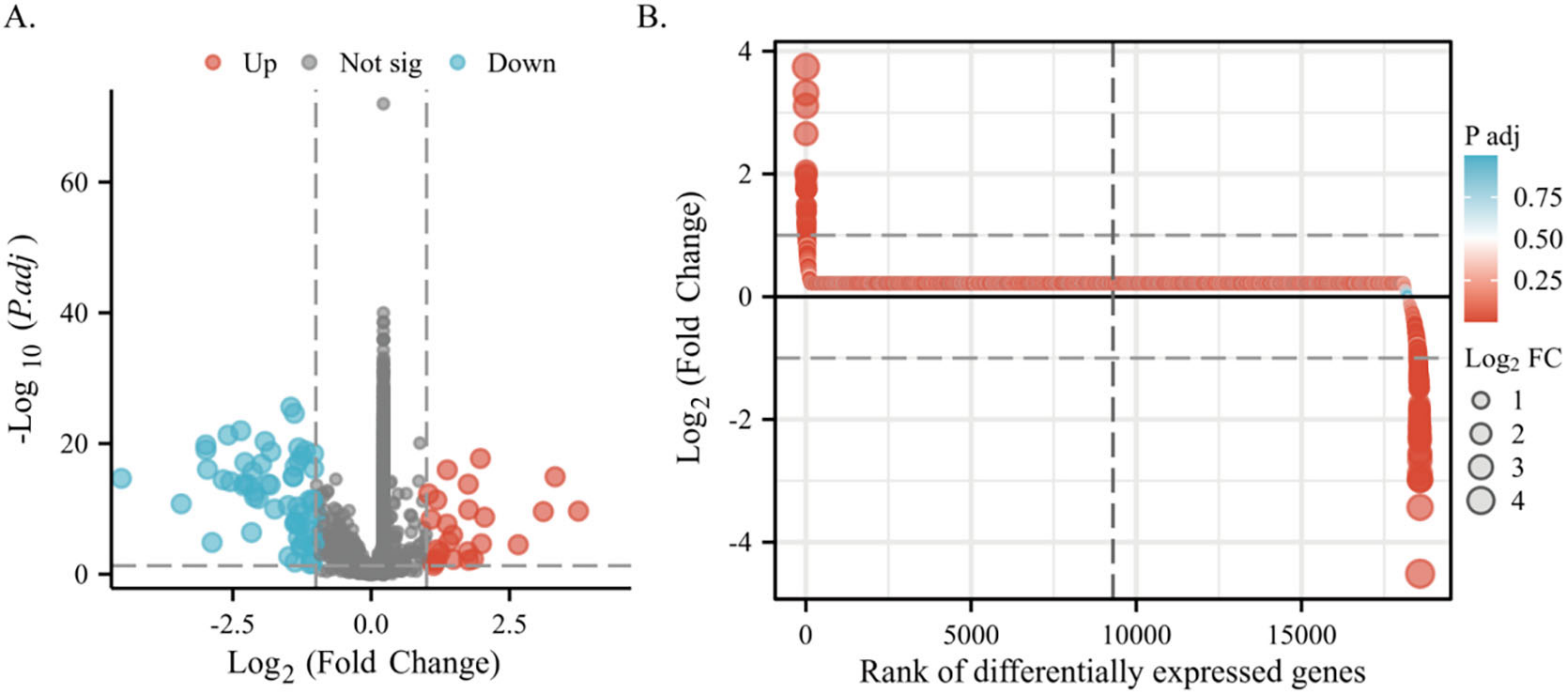
Differential gene expression associated with PSMC4 in lung adenocarcinoma. **(A)** Volcano plot displaying detailed DEGs between high and low PSMC4 expression lung adenocarcinoma groups. **(B)** Differential heatmap further illustrates the expression differences of these genes.

We stratified 539 lung adenocarcinoma patients into groups exhibiting high and low expression of PSMC4 based on the median PSMC4 expression. This stratification enabled a comprehensive analysis of differentially expressed genes (DEGs) between these cohorts. In the high PSMC4 expression group, 86 genes were identified as significantly differentially expressed (p<0.05, |log2 fold change (FC)| ≥ 1), including 26 upregulated and 60 downregulated genes (**Figures 6A, B**). A functional analysis of these genes will be detailed subsequently.

### 3.5 Functional annotation of differentially expressed genes associated with PSMC4

**Figure 7:**
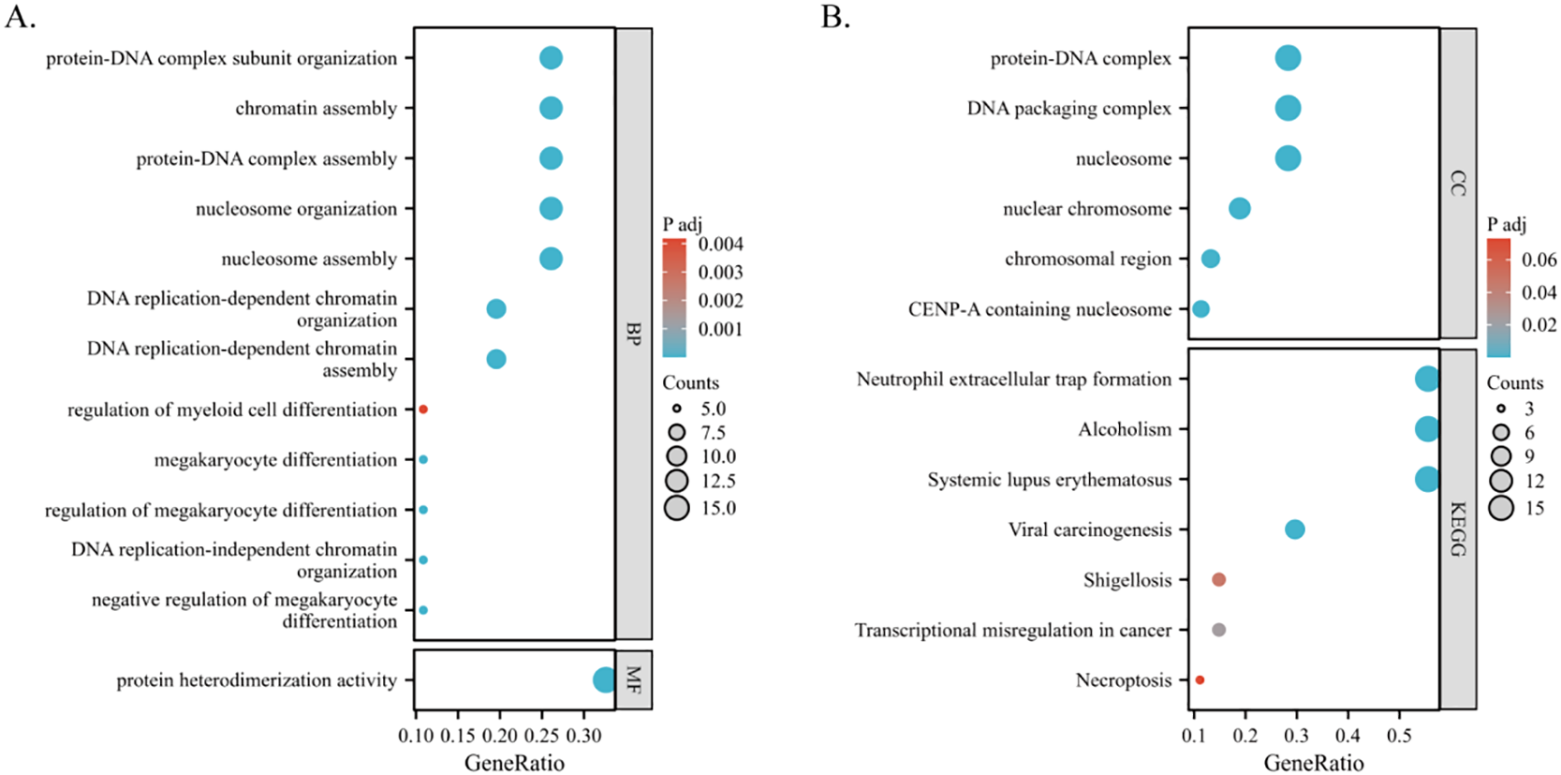
Functional enrichment analysis of PSMC4-related differentially expressed genes in lung adenocarcinoma. **(A, B)** Biological functions and biological pathways associated with PSMC4 expression-related genes were studied using GO and KEGG pathway analysis.

A thorough examination using the “ClusterProfiler” R package uncovered the functional roles of 86 differentially expressed genes across various biological processes (BP). These encompass crucial activities such as DNA replication-dependent chromatin assembly, nucleosome organization, negative regulation of megakaryocyte differentiation, and protein-DNA complex assembly, among others (**Figure 7A**). Moreover, these genes demonstrate molecular functions primarily characterized by protein heterodimerization activity (**Figure 7A**). Regarding their cellular localization, they are prominently situated within structures like the nucleosome, DNA packaging complex, and nuclear chromosome (**Figure 7B**). Notably, KEGG pathway analysis shed light on their involvement in diverse pathways including neutrophil extracellular trap formation, viral carcinogenesis, transcriptional misregulation in cancer, shigellosis, and necroptosis, underlining their significance in disease processes (**Figure 7B**).

**Figure 8:**
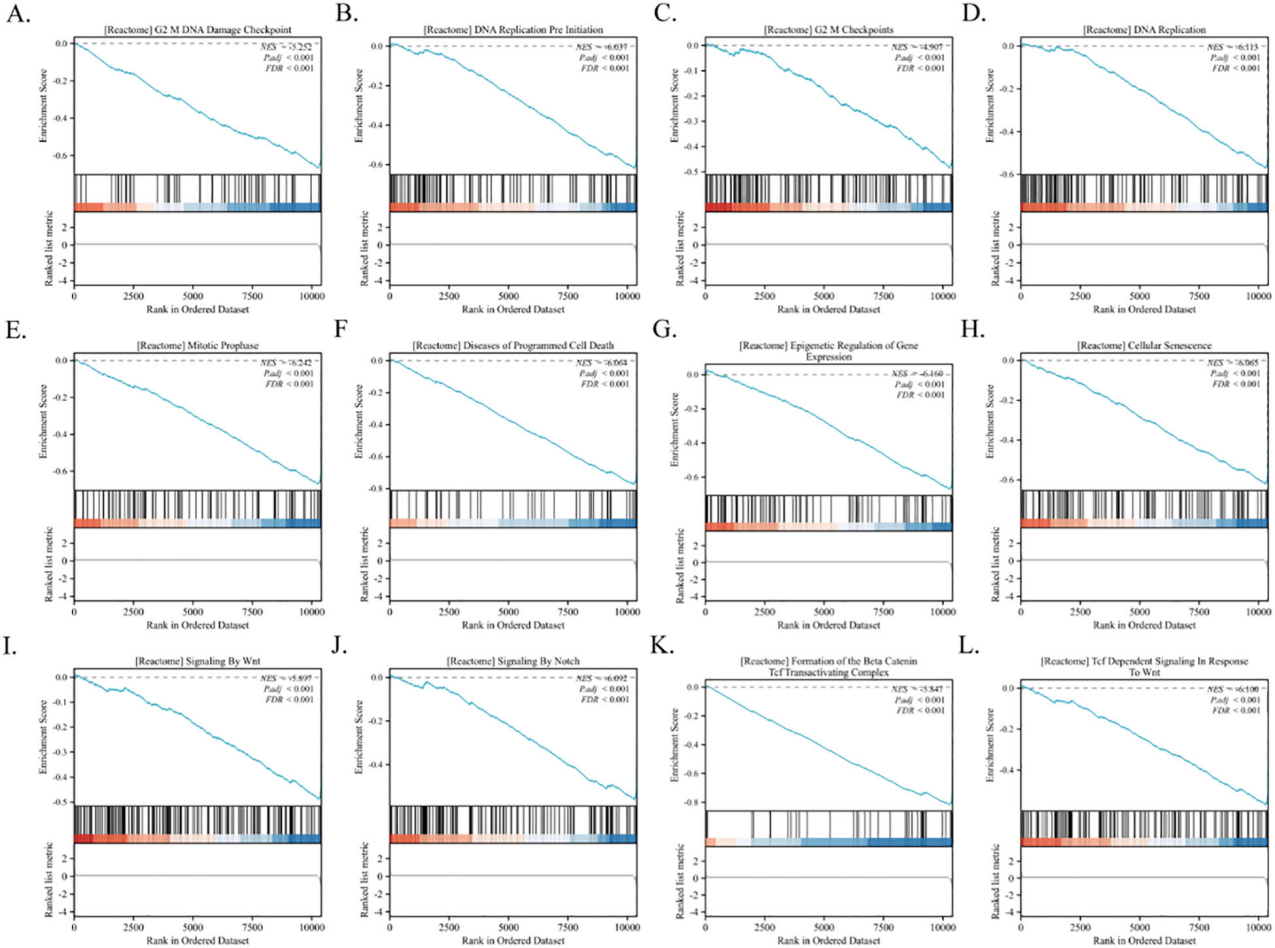
Identification of PSMC4-related signaling pathways in lung adenocarcinoma. **(A-L)** GSEA was conducted to explore the biological functions and potential signaling pathways of differentially expressed genes related to PSMC4 in lung adenocarcinoma.

Furthermore, GSEA revealed that PSMC4 is intricately linked to key biological pathways. These include the G2/M DNA Damage Checkpoint, G2/M Checkpoints, Mitotic Prophase, Epigenetic Regulation of Gene Expression, DNA Replication, Signaling by Notch, Cellular Senescence, Signaling by WNT, Formation of the Beta-Catenin TCF Transactivating Complex, Diseases of Programmed Cell Death, DNA Replication Pre-Initiation, and TCF Dependent Signaling in Response to WNT (**Figures 8A–L**). These insights are critical for advancing our understanding of the molecular dynamics associated with PSMC4 in the progression of lung adenocarcinoma.

### 3.6 Correlation between PSMC4 expression and immune cell infiltration in lung adenocarcinoma

**Figure 9:**
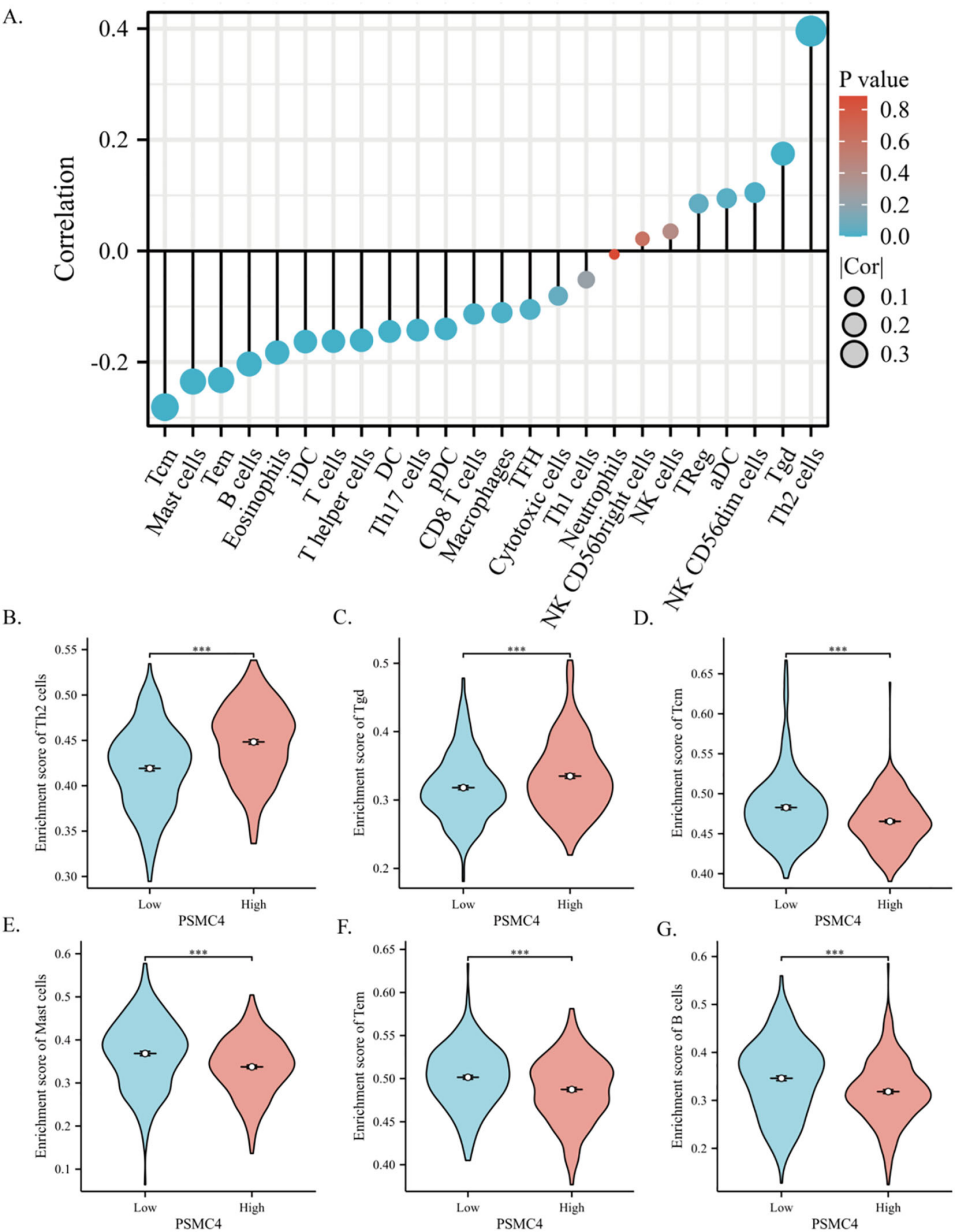
Correlation of PSMC4 with the immune microenvironment in lung adenocarcinoma. **(A)** Spearman correlation analysis of PSMC4 expression levels with the infiltration levels of 24 immune cells in lung adenocarcinoma. **(B–G)** Comparison of immune cell infiltration levels between PSMC4 high expression group and low expression group in Th2 cells, Tgd, Tcm, Mast cells, Tem, and B cells. (***p<0.001).

Utilizing ssGSEA, our study assessed the presence of 24 immune cell types within lung adenocarcinoma tissues, concurrently exploring the relationship between PSMC4 expression and immune cell infiltration using Spearman correlation analysis. Our findings underscore a marked linkage of PSMC4 expression with various immune cell subtypes. Notably, PSMC4 expression correlated positively with Th2 cells (R=0.396, p<0.001) and gamma delta T cells (R=0.175, p<0.001), but negatively with central memory T cells (Tcm) (R=-0.281, p<0.001), mast cells (R=-0.235, p<0.001), effector memory T cells (Tem) (R=-0.232, p<0.001), and B cells (R=-0.203, p<0.001). Subsequently, we verified the infiltration levels of the six most correlated immune cell types, affirming the consistency of our initial analysis (**Figures 9A–G**).

### 3.7 Validation of PSMC4 expression in non-small cell lung cancer tissues

**Figure 10:**
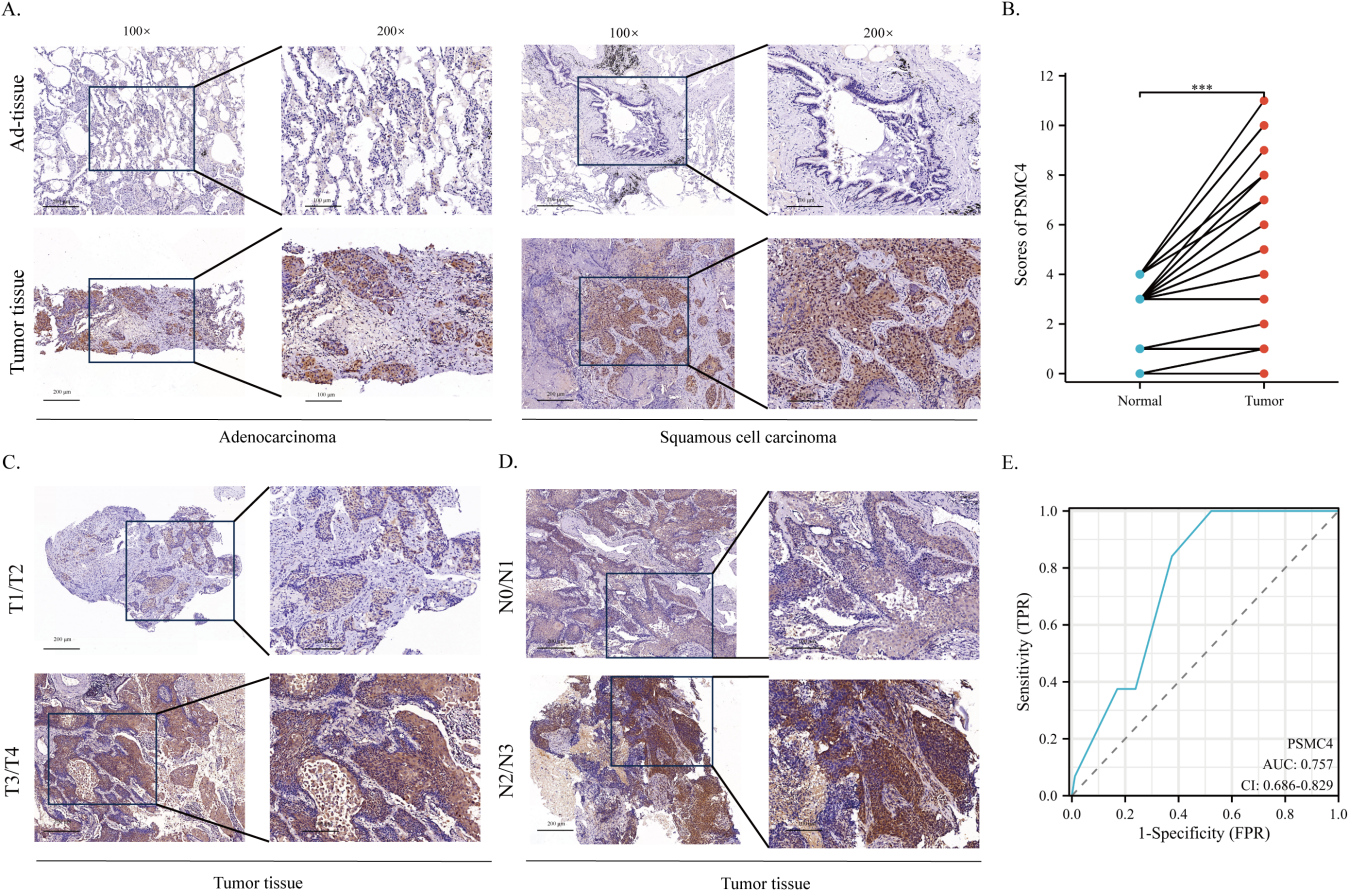
Expression of PSMC4 in non-small cell lung cancer tissues. **(A)** Representative immunohistochemical staining images illustrating PSMC4 expression in adenocarcinoma, squamous cell carcinoma, and normal lung tissues. The enlarged regions of interest are shown in the right panels (scale bars: 100 μm, 200 μm). **(B)** Quantitative analysis revealed significantly elevated PSMC4 protein levels in non-small cell lung cancer tissues (***p < 0.001). **(C)** Representative immunohistochemical staining images showing PSMC4 expression in NSCLC at T1–T2 and T3–T4 stages, with the magnified views of the rectangular regions shown on the right (scale bars: 100 μm, 200 μm). **(D)** Representative immunohistochemical staining images showing PSMC4 expression in NSCLC at N0–N1 and N2–N3 stages, with the magnified views of the rectangular regions shown on the right (scale bars: 100 μm, 200 μm). **(E)** ROC curve evaluation of the diagnostic value of PSMC4 in NSCLC.

We procured tumor and adjacent normal tissue samples from 88 individuals diagnosed with NSCLC and scrutinized PSMC4 expression and its subcellular distribution using immunohistochemistry. The analysis demonstrated a predominant cytoplasmic localization of PSMC4 (**Figure 10A**) and a significant upregulation in NSCLC tissues compared to adjacent normal tissues (p<0.05) (**Figure 10B**). Stratification of patients into high and low PSMC4 expression groups unveiled significant associations of elevated PSMC4 expression with advanced clinical T stage (p=0.031) and N stage (p=0.027) (**Table 4**). Notably, patients with NSCLC at T1–T2 stages exhibited lower PSMC4 expression compared to those at T3–T4 stages (**Figure 10C**), and a similar trend was observed for N0–N1 stage patients, who showed reduced PSMC4 expression relative to those at N2–N3 stages (**Figure 10D**). Furthermore, the diagnostic efficacy of PSMC4 in non-small cell lung cancer was evaluated through ROC curve analysis. The results indicated an AUC of 0.757, reflecting a high level of diagnostic sensitivity and specificity (**Figure 10E**).

**Table 4:** Immunohistochemical analysis of PSMC4 expression and its correlation with clinicopathological features in non-small cell lung cancer.

Characteristics	Low expression	High expression	*p*-value
n	46	42	
Gender, n (%)			0.185
Male	37 (80.4%)	38 (90.5%)	
Female	9 (19.6%)	4 (9.5%)	
Age, mean ± sd	62.478 ± 7.7194	63.405 ± 7.1774	0.562
Clinical T stage, n (%)			0.031
T1–T2	27 (58.7%)	15 (35.7%)	
T3–T4	19 (41.3%)	27 (64.3%)	
Clinical N stage, n (%)			0.027
N0-N1	25 (54.3%)	13 (31%)	
N2–N3	21 (45.7%)	29 (69%)	
Clinical M stage, n (%)			0.853
M0	25 (54.3%)	22 (52.4%)	
M1	21 (45.7%)	20 (47.6%)	
Clinical stage, n (%)			0.532
I-II	16 (34.8%)	12 (28.6%)	
III–IV	30 (65.2%)	30 (71.4%)	

### 3.8 Impact of PSMC4 on lung cancer cell proliferation

**Figure 11:**
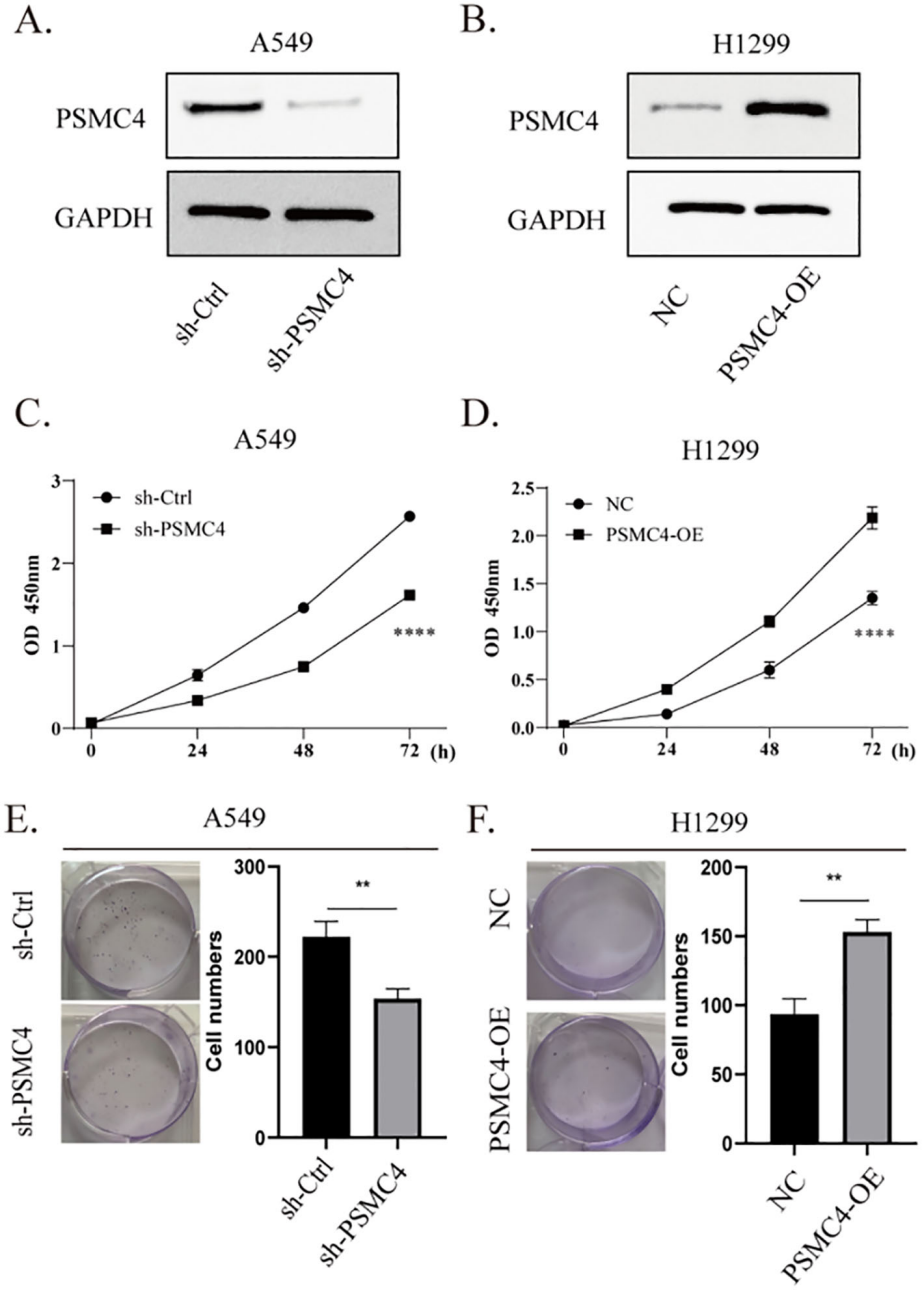
Promoting effect of PSMC4 on lung cancer cell proliferation. **(A)** Downregulation of PSMC4 expression in A549 cells. **(B)** Overexpression of PSMC4 in H1299 cells. **(C, D)** Evaluation of the impact of PSMC4 on cell proliferation by CCK-8 analysis. **(E, F)** Further investigation of the promoting effect of PSMC4 on cell growth through colony formation assay (**p<0.01, ****p<0.0001).

Functional assays were initiated by silencing PSMC4 in A549 cells and enhancing its expression in H1299 cells (**Figures 11A, B**). Relative to control groups, suppression of PSMC4 substantially reduced the viability of A549 cells (p<0.0001) (**Figure 11C**), while its overexpression significantly increased the viability of H1299 cells (p<0.0001) (**Figure 11D**). Moreover, colony formation assays aligned with these findings, further validating PSMC4’s pivotal role in modulating lung cancer cell proliferation (**Figures 11E, F**).

### 3.9 Effect of PSMC4 overexpression on tumor growth in mouse xenograft models

**Figure 12:**
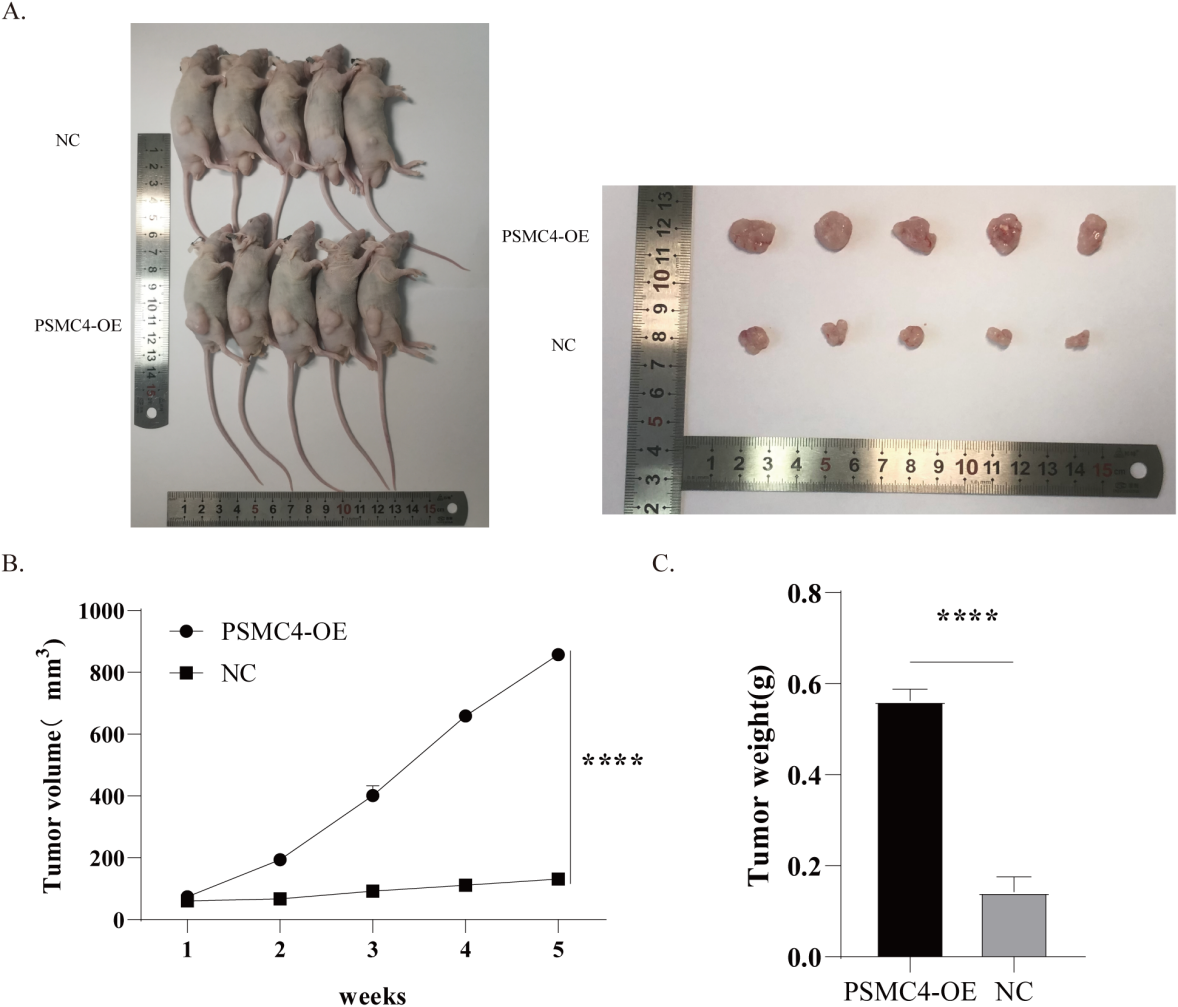
Enhanced tumor growth by overexpression of PSMC4 in mice xenografts. **(A)** Tumor samples after subcutaneous implantation of transfected H1299 cells in SCID mice for 5 weeks. **(B)** Tumor growth curve. **(C)** Comparison of tumor weights between the NC group and PSMC4 overexpression group. (****p<0.0001).

Using a subcutaneous xenograft model in mice, H1299 cells with upregulated PSMC4 exhibited tumors of significantly greater volume and weight than those in the control group (**Figures 12A–C**). These results underscore the potential of PSMC4 to facilitate tumor growth *in vivo*.

## 4 Discussion

In this investigation, we explored the role of the PSMC4 gene, a critical player in protein degradation and cell cycle regulation (21, 22), in LUAD. Our comprehensive analysis encompassed PSMC4 expression profiling in LUAD, alongside its associations with clinical-pathological features, immune cell infiltration, and functional pathways. Our findings reveal that elevated PSMC4 expression correlates significantly with more advanced T stages, N stages, and overall pathological stages. It also shows specific patterns of immune cell infiltration, underscoring its potential as both a diagnostic and prognostic biomarker. Moreover, our data highlight PSMC4’s involvement in crucial biological pathways that facilitate tumor progression and immune escape, such as the G2/M DNA damage checkpoint, Notch signaling, neutrophil extracellular trap formation, and the negative regulation of megakaryocyte differentiation. We further delineated PSMC4’s functional roles through *in vitro* and *in vivo* studies on lung cancer cells, suggesting its utility in crafting targeted and personalized treatments that could enhance the prognosis of LUAD patients. These insights not only deepen our understanding of LUAD pathogenesis but also suggest new avenues for therapeutic intervention, potentially transforming patient management and outcomes.

PSMC4, a subunit of the 26S proteasome, is integral to protein degradation and the maintenance of cellular homeostasis (23). Through meticulous analysis of the TCGA and GTEx databases, we noted the ubiquitous overexpression of PSMC4 across various cancers, particularly LUAD. This consistent overexpression highlights PSMC4’s potential as a robust biomarker for oncological assessments. Previous research across breast and prostate cancers suggests that elevated PSMC4 levels are closely linked with tumor progression (10, 14). We extend these findings to LUAD, showing that high PSMC4 expression correlates significantly with increased T stage, N stage, and overall pathological stage, indicative of its role in promoting tumor aggressiveness and metastatic capacity. Our statistical analyses confirm the relationship between PSMC4 overexpression and advanced pathological stages, suggesting its involvement in facilitating local invasion and lymph node metastasis. Additionally, we utilized ROC curve analysis to assess PSMC4’s diagnostic capabilities in LUAD. The AUC demonstrates high diagnostic sensitivity and specificity, affirming PSMC4’s utility as an effective diagnostic biomarker. Survival analyses further reveal that LUAD patients with elevated PSMC4 expression experience significantly reduced OS, DSS, and PFS relative to those with lower expression levels. These results align with and extend previous findings (14), underscoring PSMC4’s potential not just as a diagnostic tool but also as a predictor of clinical outcomes in cancer therapy.

The enriched analysis of PSMC4-associated differentially expressed genes, as detailed through comprehensive GO and KEGG pathways, significantly enhances our understanding of the molecular mechanisms by which PSMC4 influences lung adenocarcinoma progression. Notably, the GO analysis highlights the pivotal role of these genes in fundamental biological processes such as DNA replication-dependent and independent chromatin organization, protein-DNA complex assembly, and regulation of megakaryocyte differentiation. Such involvement underscores the influence of PSMC4 on chromatin dynamics, which is critical for cellular proliferation and genome stability-key elements in tumorigenesis (24, 25). Furthermore, the KEGG pathway analysis elucidates the association of these genes with several cancer-related pathways, including transcriptional misregulation in cancer and viral carcinogenesis, pointing to a broader impact of PSMC4 on oncogenic processes. Importantly, the GSEA reveals that PSMC4 is significantly linked with pathways like the G2/M DNA damage checkpoint and mitotic prophase, which are crucial for cell cycle control and the maintenance of chromosomal integrity during cell division (26, 27). The association with pathways such as signaling by NOTCH and WNT, as well as the formation of the beta-catenin TCF transactivating complex, further suggests a role for PSMC4 in signal transduction mechanisms that govern cellular differentiation, proliferation, and apoptosis. These insights are consistent with findings from previous studies, which have established the involvement of these pathways in the regulation of apoptosis and cell cycle progression in various cancers. For instance, disruptions in the NOTCH signaling pathway have been linked to a variety of cancers, including lung adenocarcinoma, by influencing cell fate decisions and maintaining the balance between cell proliferation and death (28, 29). Additionally, aberrant WNT signaling has been implicated in tumorigenesis through its effects on cellular senescence and differentiation (30, 31) By correlating these pathways with PSMC4 activity, our study not only confirms the gene’s pivotal role in cellular regulatory mechanisms but also highlights potential therapeutic targets. Modulating PSMC4 expression or its downstream pathways could offer new strategies for the treatment of lung adenocarcinoma, a prospect that warrants further investigation.

Within the immune environment of LUAD, the expression of PSMC4 is closely linked with the infiltration of specific immune cell subsets, underscoring its potential influence on tumor immune dynamics. Employing ssGSEA, we quantified the presence of 24 distinct immune cell types in LUAD tissues. Our Spearman correlation analysis demonstrated a significant association between PSMC4 expression and various immune cell subtypes, indicative of PSMC4’s crucial role in the immune evasion strategies characteristic of LUAD. Notably, increased PSMC4 expression correlates positively with the presence of Th2 cells and γδ T cells (Tgd). This relationship may skew the immune milieu of LUAD towards either a pro-inflammatory or immunosuppressive state through the enhanced recruitment of these cells. Typically, Th2 cell activity is linked with a diminished anti-tumor response and heightened inflammatory conditions (32, 33), consistent with the pro-tumorigenic influence of PSMC4. Conversely, although γδ T cells can exert anti-tumor effects under certain conditions, their presence may also promote tumor growth and facilitate immune escape in other scenarios (34, 35). Conversely, PSMC4 expression inversely correlates with Tcm, mast cells, Tem, and B cells. These cells are generally pivotal to robust anti-tumor immune responses. Thus, lower levels of PSMC4 could favor the accumulation of these beneficial immune cell types, potentially curtailing tumor growth. Particularly, a decrease in central memory T cells and effector memory T cells might compromise sustained and long-term anti-tumor immunity, exacerbating tumor immune evasion (36–39).

Our immunohistochemical analysis has revealed a significant upregulation of PSMC4 expression in NSCLC tissues compared to adjacent normal tissues. The cytoplasmic localization of PSMC4 in cancer cells indicates its involvement in proteasomal degradation pathways, which are commonly utilized by cancer cells to promote survival and proliferation (40, 41). The correlation between high PSMC4 expression and advanced clinical T and N stages further emphasizes its role in driving cancer aggressiveness. These findings are in line with previous studies (42), underscoring the crucial role of proteasome subunits in supporting cancer cell growth and survival. Therefore, targeting PSMC4 and the proteasome pathway may present novel therapeutic opportunities for NSCLC patients. Functional analysis has demonstrated that PSMC4 significantly impacts the proliferation and growth of lung cancer cells. Knockdown of PSMC4 in A549 cells resulted in reduced cell viability and colony formation, while its overexpression in H1299 cells enhanced these properties. These results indicate that PSMC4 functions as an oncogene in lung adenocarcinoma, promoting cell proliferation and tumorigenesis. This is consistent with previous research showing that PSMC4 knockout inhibits proliferation, cell cycle progression, and *in vivo* migration of prostate cancer cells while inducing apoptosis (14). *In vivo* xenograft models have further validated these findings, showing that PSMC4 overexpression leads to increased tumor volume and weight. In our experiments, although PSMC4-knockout A549 cells and PSMC4-overexpressing H1299 cells showed only a 1.5-fold proliferation difference *in vitro*, this discrepancy increased 3- to 8-fold in immunodeficient mouse models. Several factors may explain this: PSMC4 could enhance tumor growth by modulating tumor microenvironmental cells, such as immune and endothelial cells, promoting angiogenesis, and recruiting fibroblasts. Additionally, the longer duration in xenograft models allows for metastasis and angiogenesis, and PSMC4 might aid immune evasion, further promoting tumor growth in the absence of functional immune surveillance. These functional insights highlight the potential of PSMC4 as a therapeutic target, suggesting that inhibiting its expression or function could impede the progression of lung cancer.

Despite the comprehensive approach and robust methodologies employed in this study, several limitations warrant acknowledgment. First, the research predominantly relies on bioinformatics analyses and lacks extensive wet lab validation, which could provide more definitive evidence regarding the role of PSMC4 in lung adenocarcinoma. Second, the sample size—particularly for clinical validation—is relatively small, potentially constraining the generalizability of the findings. Furthermore, the absence of a thorough clinical validation analysis is a significant limitation, as such analysis is crucial for translating these findings into clinical practice.

In conclusion, this study offers valuable insights into the expression of the PSMC4 gene in lung adenocarcinoma and its association with clinical pathological features, immune cell infiltration, and functional enrichment. The findings suggest that PSMC4 may serve as a promising diagnostic biomarker and therapeutic target for lung adenocarcinoma. Future research should prioritize larger, more diverse cohorts and incorporate extensive wet lab validation to corroborate these findings. Additionally, integrating clinical validation analyses will be essential for translating these discoveries into practical clinical applications. The results of this study lay the groundwork for further exploration of PSMC4’s role in cancer biology and its potential to enhance the diagnosis and treatment of lung adenocarcinoma.

## Data Availability

The raw data supporting the conclusions of this article will be made available by the authors, without undue reservation.
